# Role of surgery in T4N0-3M0 esophageal cancer

**DOI:** 10.1186/s12957-023-03239-8

**Published:** 2023-11-27

**Authors:** Chen Qi, Liwen Hu, Chi Zhang, Kang Wang, Bingmei Qiu, Jun Yi, Yi Shen

**Affiliations:** 1 Department of Cardiothoracic Surgery, Jinling Hospital, Medical School of Nanjing University, Nanjing, 210002 China; 2 Department of Cardiothoracic Surgery, Jinling Hospital, Jinling Clinical Medical School, Nanjing Medical University, Nanjing, 210002 China; 3 Department of Anesthesiology, Women’s Hospital of Nanjing Medical University, Nanjing Maternity and Child Health Care Hospital, Nanjing, 210004 China

**Keywords:** T4, Esophageal cancer, Surgery, PSM, SEER

## Abstract

**Background:**

This study aimed to investigate an unsettled issue that whether T4 esophageal cancer could benefit from surgery.

**Methods:**

Patients with T4N0-3M0 esophageal cancer from 2004 to 2015 from the Surveillance, Epidemiology, and End Results (SEER) database were included in this study. Kaplan–Meier method, Cox proportional hazard regression, and propensity score matching (PSM) were used to compare overall survival (OS) between the surgery and no-surgery group.

**Results:**

A total of 1822 patients were analyzed. The multivariable Cox regression showed the HR (95% CI) for surgery vs. no surgery was 0.492 (0.427–0.567) (*P* < 0.001) in T4N0-3M0 cohort, 0.471 (0.354–0.627) (*P* < 0.001) in T4aN0-3M0 cohort, and 0.480 (0.335–0.689) (*P* < 0.001) in T4bN0-3M0 cohort. The HR (95% CI) for neoadjuvant therapy plus surgery vs. no surgery and surgery without neoadjuvant therapy vs. no surgery were 0.548 (0.461–0.650) (*P* < 0.001) and 0.464 (0.375–0.574) (*P* < 0.001), respectively. No significant OS difference was observed between neoadjuvant therapy plus surgery and surgery without neoadjuvant therapy: 0.966 (0.686–1.360) (*P* = 0.843). Subgroup analyses and PSM-adjusted analyses showed consistent results.

**Conclusion:**

Surgery might bring OS improvement for T4N0-3M0 esophageal cancer patients, no matter in T4a disease or in T4b disease. Surgery with and without neoadjuvant therapy might both achieve better OS than no surgery.

**Supplementary Information:**

The online version contains supplementary material available at 10.1186/s12957-023-03239-8.

## Introduction

Esophageal cancer accounts for 3.1% of all newly diagnosed cancers and the 5-year overall survival (OS) is approximately 30% [1, 2]. As an aggressive malignant tumor, it is often diagnosed at a late stage. The esophagus has the characteristics which are lack of serosa and being closely encompassed by adjacent organs including the trachea, bronchus, heart, and large vascular vessels, so it is easy for esophageal cancer to grow through the esophageal wall and invade the adjacent organs [3]. The invasion of adjacent organs is classified as T4 according to the 8th TNM staging system, and this population has a bad prognosis [4, 5]. As written in the NCCN guidelines for esophageal and esophagogastric junction cancers, definitive chemoradiotherapy or induction chemoradiotherapy plus surgery are recommended for T4a esophageal cancer, while only definitive chemoradiotherapy is recommended for T4b disease except that chemotherapy alone is recommended for the setting of invasion of the trachea, heart, great vessels, or vertebral body [4].

The milestone randomized controlled trials (RCTs), RTOG 85–01 trial and CROSS trial validated the efficacy of chemoradiotherapy alone and chemoradiotherapy plus surgery for locally advanced esophageal cancer, respectively [6, 7]. However, neither of those trials included patients with T4 disease. In spite of the recommendation of definitive chemoradiotherapy for T4 esophageal cancer, the proportion of clinical complete response is only 25–32% [8]. Some prospective studies have been reported to show the benefit of chemoradiotherapy alone or chemoradiotherapy plus surgery in those patients with T4 disease [9–11]. One of those studies demonstrated that the chemoradiotherapy plus surgery group and chemoradiotherapy alone group had a 5-year OS of 17% and 13%, respectively, indicating a tendency favoring the surgery group but with no statistical significance [10]. These studies provided limited evidence because of their non-randomized nature. However, no RCT referring to the identification of the role of esophagectomy in T4 disease was reported. Two retrospective studies regarding to comparison of surgery vs. no surgery for T4 esophageal cancer were performed [12, 13]. Both studies revealed no significant OS difference between esophagectomy and no surgery group. Another study suggested some unresectable T4 disease could be resected after induction chemotherapy or chemoradiotherapy, and some had comparable 5-year OS to those immediately resected T4 disease on condition that R0 resection could be achieved [14].

As the role of surgery in T4 esophageal cancer is still unclear, we downloaded data of patients with T4N0-3M0 (8th version) esophageal cancer and conducted this multicenter study to compare the OS outcome between the surgery group and the surgery group.

## Methods

### Study cohort

All the data was extracted from the Surveillance, Epidemiology, and End Results (SEER) database which was constructed by the National Cancer Institute (https://seer.cancer.gov/) and contained information on cancer of about 28% of the population of the USA. A subset of data submitted by 17 cancer registries was used in the extraction. After transforming the 6th or 7th version American Joint Committee on Cancer (AJCC) TNM staging system into the 8th version, patients diagnosed with AJCC 8th T4N0-3M0 esophageal cancer (only one primary) from January 2004 to December 2015 were included for screening. The exclusion criteria are as follows: patients who were aged < 18 years, diagnosed with autopsy/death certificate without pathological confirmation, whether received surgery or not was not known. The esophageal cancer was recognized using the “Site recode the 3rd edition of International Classification of Disease for Oncology (ICD-O-3)” of “Esophagus”. The histological types were classified based on ICD-O-3 classification: adenocarcinoma (8140, 8141, 8143–8145, 8190–8231, 8260–8263, 8310, 8401, 8480–8490, 8550, 8551, 8570–8574, and 8576), squamous cell carcinoma (8050–8078, 8083, and 8084), and other histological types [15]. Ethical approval and informed consent were exempt from review by the institutional review board because all data from the SEER database was deidentified and publicly accessible. The study was conducted in accordance with the revised version of the Declaration of Helsinki.

### Variables and endpoint

The variables used in the study were as follows: year of diagnosis, age, sex, race, primary site (location of tumor), histology, differentiation, T stage, N stage, neoadjuvant therapy, regional nodes examined, radiotherapy, and chemotherapy. Neoadjuvant therapy was defined as neoadjuvant systemic therapy, radiotherapy, or both. The endpoint was set as OS, defined as the time from diagnosis to all-cause death reported with the unit of the month. The patients were followed until December 2019.

### Statistical analysis

In the comparison between the no surgery and surgery group, first, the variables, year of diagnosis, age, sex, race, primary site, histology, differentiation, T stage, N stage, radiotherapy, and chemotherapy were put into the univariable Cox proportional hazard regression model. Second, the variables with *P* < 0.1 were entered into the multivariable Cox regression analysis. To better balance the clinicopathologic features of the two groups, propensity score matching was performed using a multivariable Logistic regression model. The aforementioned variables were put into the multivariable Logistic regression model to calculate the propensity score and generate matched samples with a caliper of 0.2 and a ratio of 2:1 for the surgery group vs. surgery group. The balance of baseline features was measured by the standardized mean difference (SMD), and a value of SMD < 0.1 was deemed as a good balance. Kaplan–Meier curve with log-rank test was also conducted in comparison before and after PSM. Sensitivity analysis comparing surgery vs. no surgery in stage T4aN0-3M0 and T4bN0-3M0 separately were also performed with Kaplan–Meier curve before and after propensity score matching (PSM), univariable/multivariable Cox regression analysis, and PSM-adjusted univariable Cox regression analysis, with multivariable analysis adjusting the aforementioned parameters. Besides, the impact of neoadjuvant therapy surgery vs. no surgery and surgery without neoadjuvant therapy vs. no surgery in the study cohort were also explored using the same analytic strategy. In addition, neoadjuvant therapy plus surgery was also compared with surgery without neoadjuvant therapy using the same analytic strategy except the variable regional nodes examined were adjusted in the analysis. The effect of surgery vs. no surgery was also explored in esophageal adenocarcinoma and esophageal squamous cell carcinoma with known TNM stage (IIIB-IVA) separately in sensitivity analyses.

Data was downloaded using the SEER*Stat software version 8.4.0 (https://seer.cancer.gov/). All the statistical analyses were carried out using R software version 4.2.0 (https://www.r-project.org/). Statistical significance was defined as two-sided *P* < 0.05.

## Results

### Patient features

As shown in Supplementary Table S1, a total of 1,826 patients with 8th AJCC T4N0-3M0 (only one primary) esophageal cancer from January 2004 to December 2015 were screened and only 4 patients without surgery information were excluded, so 1822 samples remained for analysis. All the baseline features had an SMD > 0.2 indicating a significant difference, and the surgery group tended to have more patients diagnosed in 2004–2009, aged < 65 years, being male, being white, with tumor located in the lower third of the esophagus, with adenocarcinoma, with lymph node metastasis, received radiotherapy/chemotherapy (Table 1). After PSM, the baseline features of the two groups were well balanced with all SMDs smaller than or very close to 0.1 (Supplementary Figure S1A).

**Table 1:** Baseline characteristics of no surgery cohort and surgery cohort of AJCC 8th T4N0-3M0 EC patients

Variables	No surgery *n* = 1478	Surgery *n* = 344	SMD
Year of diagnosis			0.210
2004–2009	789 (53.4%)	219 (63.7%)	
2010–2015	689 (46.6%)	125 (36.3%)	
Age			0.253
< 65 years old	691 (46.8%)	204 (59.3%)	
> = 65 years old	787 (53.2%)	140 (40.7%)	
Sex			0.216
Male	1104 (74.7%)	287 (83.4%)	
Female	374 (25.3%)	57 (16.6%)	
Race			0.367
White	1057 (71.5%)	293 (85.2%)	
Black	307 (20.8%)	29 (8.4%)	
Other/unknown	114 (7.7%)	22 (6.4%)	
Primary site			0.608
Upper third	285 (19.3%)	21 (6.1%)	
Middle third	302 (20.4%)	45 (13.1%)	
Lower third	607 (41.1%)	236 (68.6%)	
Unknown	284 (19.2%)	42 (12.2%)	
Histology			0.679
Adenocarcinoma	530 (35.9%)	233 (67.7%)	
Squamous cell carcinoma	824 (55.8%)	101 (29.4%)	
Other	124 (8.4%)	10 (2.9%)	
Differentiation			0.265
Grade I	53 (3.6%)	19 (5.5%)	
Grade II	494 (33.4%)	126 (36.6%)	
Grade III/IV	610 (41.3%)	157 (45.6%)	
Unknown	321 (21.7%)	42 (12.2%)	
T stage			0.431
T4a	318 (21.5%)	97 (28.2%)	
T4b	438 (29.6%)	43 (12.5%)	
T4, NOS	722 (48.8%)	204 (59.3%)	
*N* stage			0.568
N0	669 (45.3%)	115 (33.4%)	
N1	345 (23.3%)	109 (31.7%)	
N2	72 (4.9%)	44 (12.8%)	
N3	30 (2.0%)	32 (9.3%)	
N1–3, NOS	362 (24.5%)	44 (12.8%)	
Neoadjuvant therapy			
No	–	110 (32.0%)	
Yes	–	234 (68.0%)	
Regional nodes examined			
< 15	–	216 (62.8%)	
> = 15	–	126 (36.6%)	
Unknown	–	2 (0.6%)	
Radiotherapy			0.316
No/unknown	541 (36.6%)	77 (22.4%)	
Yes	937 (63.4%)	267 (77.6%)	
Chemotherapy			0.498
No/unknown	569 (38.5%)	58 (16.9%)	
Yes	909 (61.5%)	286 (83.1%)	

*EC* Esophageal cancer, *NOS* not otherwise specified

### Survival analysis

**Fig. 1:**
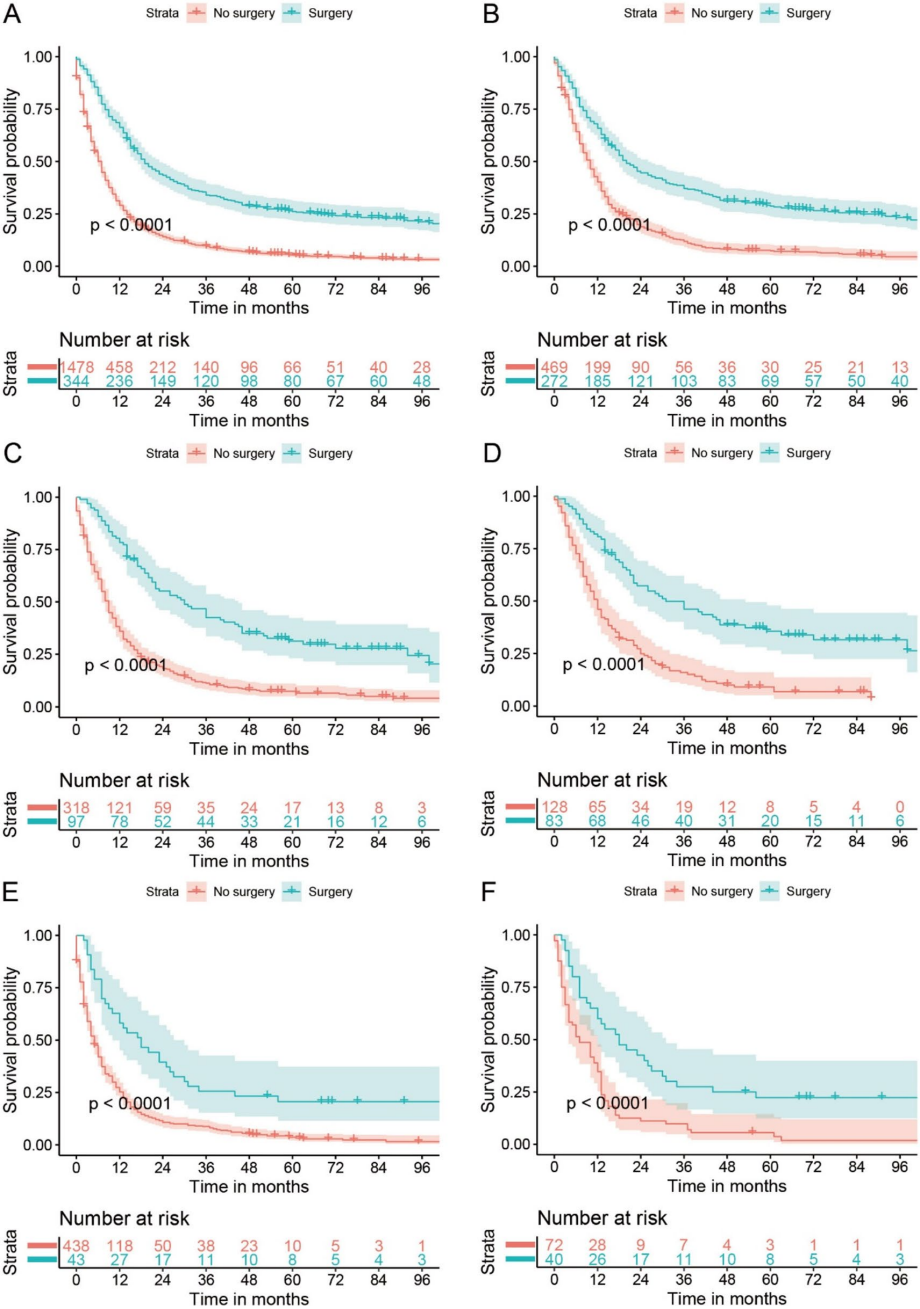
Survival curves of OS for stage T4N0-3M0 EC comparing surgery with no surgery before PSM (**A**) and after PSM (**B**), stage T4aN0-3M0 EC comparing surgery with no surgery before PSM (**C**) and after PSM (**D**), stage T4bN0-3M0 EC comparing surgery with no surgery before PSM (**E**) and after PSM (**F**). OS, overall survival; EC, esophageal cancer; PSM, propensity score matching

In the study cohort, 1412 of the no-surgery group (*n* = 1478) and 280 of the surgery group (*n* = 344) died until December 2019 with a median follow-up time (interquartile range) of 8 (3–18) months. The median OS time [95% confidence interval (CI)] of the surgery group was 7 (6–7) months, and the corresponding data of surgery group was 19 (17–23) months. The 1-, 3-, and 5-year OS rate (95% CI) of the no surgery group was 29.0% (26.8%-31.4%), 9.5% (8.1–11.1%), and 5.4% (4.3–6.7%), respectively, and the corresponding rates of the surgery group was 66.3% (61.5–71.5%), 33.9% (29.3–39.4%), and 26.1% (21.8%-31.3%), respectively. The Kaplan–Meier curves in Fig. 1A, B demonstrated that the surgery could bring better OS both before and after PSM (both log-rank *P* < 0.001).

In Table 2, the univariable Cox analysis showed surgery, age, race, primary site, histology, differentiation, T stage, N stage, radiotherapy, and chemotherapy were potential prognostic factors (*P* < 0.1) for OS of T4N0-3M0 esophageal cancer patients. After multivariable Cox analysis, all these variables remained as the independent prognostic factors except for age. In Tables 2 and 3, the multivariable analysis for surgery vs. no surgery and PSM-adjusted analysis both showed hazard ratio (HR) (95% CI) favoring surgery: 0.492 (0.427–0.567) (*P* < 0.001) and 0.493 (0.417–0.582) (*P* < 0.001).

**Table 2:** Univariable and multivariable Cox regression analysis comparing no surgery with surgery for the OS of AJCC 8th T4N0-3M0 EC patients

Variables	Univariable analysis		Multivariable analysis	
	HR (95%CI)	*P*	HR (95%CI)	*P*
Surgery				
No	1		1	
Yes	0.435 (0.382–0.496)	< 0.001	0.492 (0.427–0.567)	< 0.001
Year of diagnosis				
2004–2009	1			
2010–2015	0.967 (0.878–1.065)	0.497		
Age				
< 65 years old	1		1	
> = 65 years old	1.243 (1.130–1.368)	< 0.001	1.102 (0.998–1.217)	0.055
Sex				
Male	1			
Female	1.039 (0.928–1.163)	0.504		
Race				
White	1		1	
Black	1.375 (1.217–1.555)	< 0.001	1.060 (0.925–1.214)	0.401
Other/unknown	0.925 (0.767–1.115)	0.412	0.809 (0.668–0.980)	0.031
Primary site				
Upper third	1		1	
Middle third	1.190 (1.015–1.396)	0.033	1.283 (1.090–1.510)	0.003
Lower third	0.857 (0.747–0.982)	0.027	1.105 (0.938–1.301)	0.233
Unknown	1.260 (1.072–1.481)	0.005	1.198 (1.012–1.417)	0.035
Histology				
Adenocarcinoma	1		1	
Squamous cell carcinoma	1.408 (1.274–1.556)	< 0.001	1.347 (1.177–1.542)	< 0.001
Other	1.671 (1.383–2.021)	< 0.001	1.376 (1.128–1.678)	0.002
Differentiation				
Grade I	1		1	
Grade II	1.175 (0.911–1.517)	0.214	1.187 (0.918–1.535)	0.191
Grade III/IV	1.365 (1.060–1.756)	0.016	1.464 (1.134–1.890)	0.003
Unknown	1.296 (0.995–1.688)	0.055	1.182 (0.904–1.545)	0.221
T stage				
T4a	1		1	
T4b	1.543 (1.344–1.772)	< 0.001	1.193 (1.031–1.381)	0.018
T4, NOS	1.219 (1.079–1.378)	0.002	1.103 (0.962–1.265)	0.159
N stage				
N0	1		1	
N1	0.861 (0.763–0.972)	0.015	1.116 (0.980–1.271)	0.098
N2	0.834 (0.681–1.022)	0.08	1.084 (0.879–1.337)	0.452
N3	1.136 (0.875–1.476)	0.339	1.717 (1.310–2.250)	< 0.001
N1–3, NOS	1.071 (0.947–1.212)	0.274	1.166 (1.024–1.328)	0.021
Radiotherapy				
No/unknown	1		1	
Yes	0.433 (0.391–0.480)	< 0.001	0.573 (0.508–0.646)	< 0.001
Chemotherapy				
No/unknown	1		1	
Yes	0.368 (0.332–0.408)	< 0.001	0.497 (0.439–0.562)	< 0.001

*OS* Overall survival, *EC* Esophageal cancer, *HR* Hazard ratio, *CI* Confidential interval, *NOS* not otherwise specified

**Table 3:** Sensitivity analysis of the influence of surgery on OS of stage T4N0-3M0 EC

Variables	Before PSM				After PSM	
	Univariable analysis		Multivariable analysis			
	HR (95%CI)	*P*	HR (95%CI)	*P*	HR (95%CI)	*P*
Surgery vs. no surgery in T4	0.435 (0.382–0.496)	< 0.001	0.492 (0.427–0.567)	< 0.001	0.493 (0.417–0.582)	< 0.001
Surgery vs. no surgery in T4a	0.377 (0.289–0.493)	< 0.001	0.471 (0.354–0.627)	< 0.001	0.391 (0.282–0.543)	< 0.001
Surgery vs. no surgery in T4b	0.423 (0.297–0.603)	< 0.001	0.480 (0.335–0.689)	< 0.001	0.434 (0.282–0.67)	< 0.001
Neo + surgery vs. no surgery in T4	0.373 (0.319–0.437)	< 0.001	0.548 (0.461–0.650)	< 0.001	0.448 (0.367–0.548)	< 0.001
Surgery without neo vs. no surgery in T4	0.628 (0.512–0.769)	< 0.001	0.464 (0.375–0.574)	< 0.001	0.587 (0.435–0.792)	< 0.001
Neo + surgery vs. surgery without neo in T4	0.586 (0.459–0.748)	< 0.001	0.966 (0.686–1.360)	0.843	0.892 (0.58–1.372)	0.603
Surgery vs. no surgery in adenocarcinoma	0.373 (0.289–0.481)	< 0.001	0.432 (0.331–0.565)	< 0.001	0.435 (0.33–0.574)	< 0.001
Surgery vs. no surgery in squamous cell carcinoma	0.511 (0.349–0.749)	0.001	0.388 (0.26–0.579)	< 0.001	0.536 (0.335–0.859)	0.01

*PSM* Propensity score matching, *OS* Overall survival, *EC* Esophageal cancer, *HR* Hazard ratio, *CI* Confidential interval, *Neo* neoadjuvant therapy

### Subgroup and sensitivity analysis

**Fig. 2:**
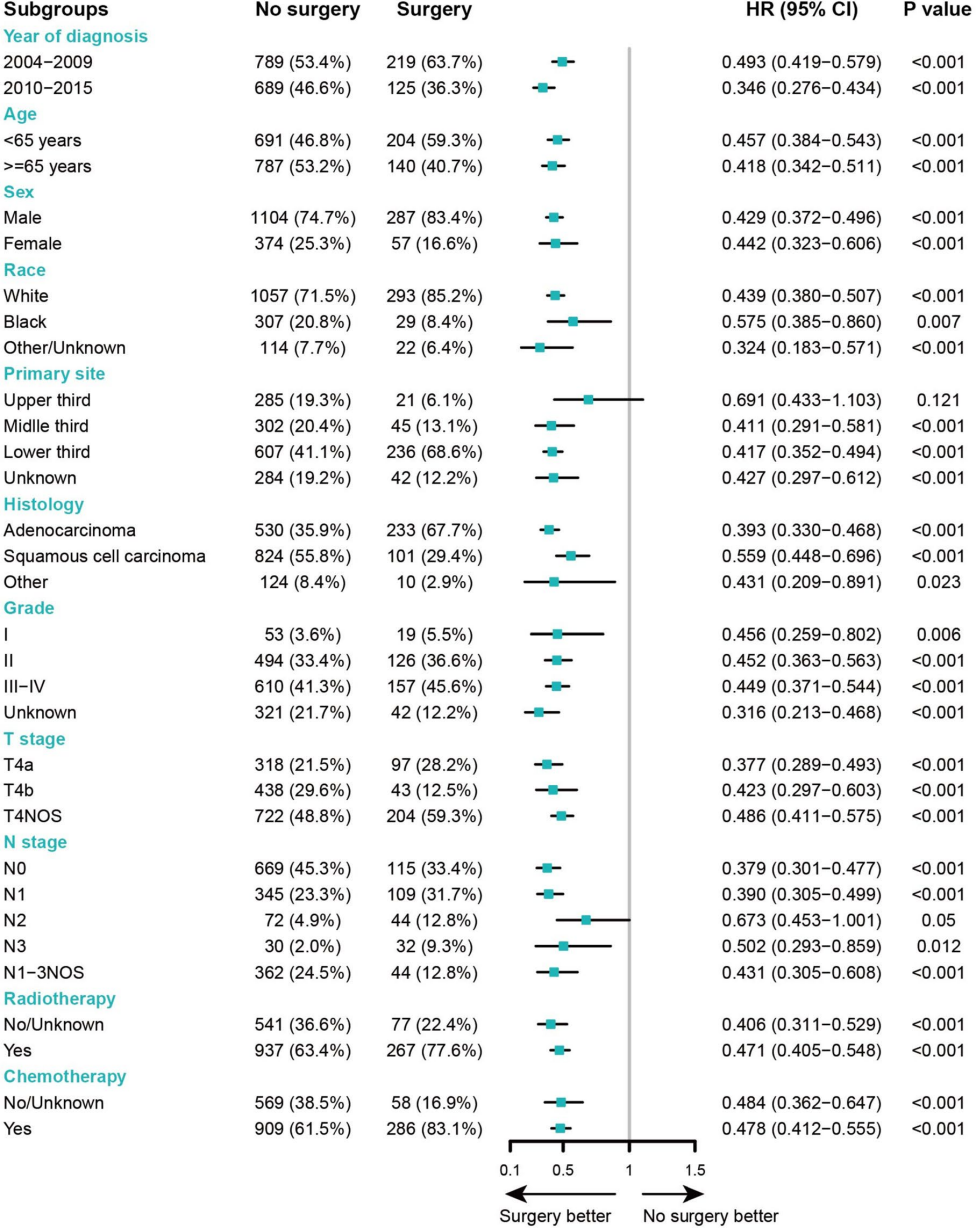
Subgroup analysis of HR for surgery vs. no surgery in OS of stage T4N0-3M0 EC. HR, hazard ratio; OS, overall survival; EC, esophageal cancer; CI, confidential interval

The forest plots of subgroup analyses showing HR (95%CI) for surgery vs. no surgery in T4N0-3M0, T4aN0-3M0, and T4bN0-3M0 cohort were presented in Fig. 2, Supplementary Figure S2, and Supplementary Figure S3, respectively. All the subgroup analyses suggested surgery group had a tendency of better OS than the surgery group except the subgroup of N2 for the T4bN0-3M0 cohort with a point estimate of HR > 1 in Supplementary Figure S3 which might be caused by the limited sample size.

**Fig. 3:**
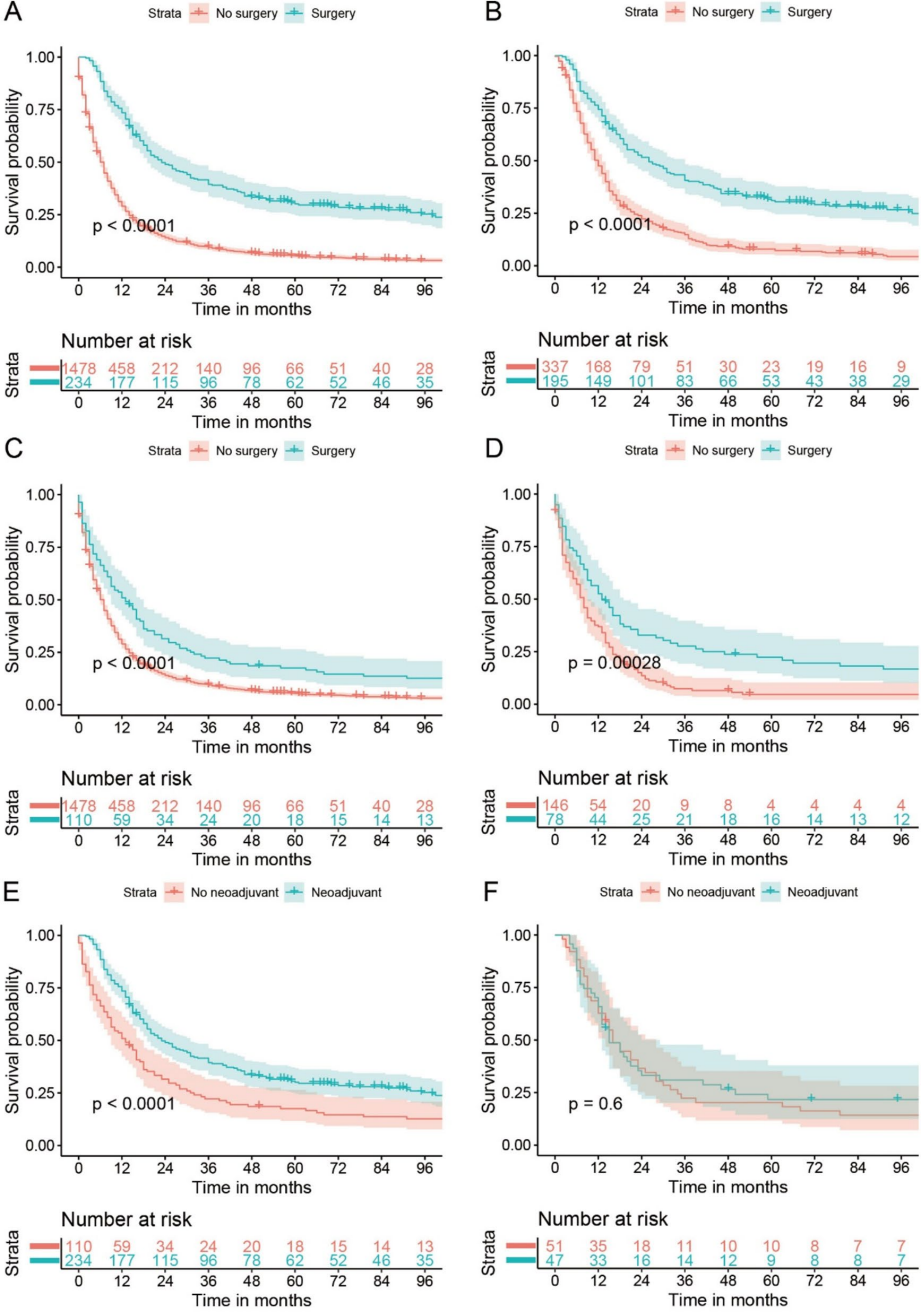
Survival curves of OS for stage T4N0-3M0 EC for Neo + surgery vs. no surgery before PSM (**A**) and after PSM (**B**), surgery without Neo vs. no surgery before PSM (**C**) and after PSM (**D**), Neo + surgery vs. surgery without Neo before PSM (**E**) and after PSM (**F**). OS, overall survival; EC, esophageal cancer; Neo, neoadjuvant therapy; PSM, propensity score matching

In the sensitivity analyses, the baseline features of surgery vs. no surgery in T4aN0-3M0 (Supplementary Figure S1B) and T4bN0-3M0 (Supplementary Figure S1C) cohort, neoadjuvant therapy plus surgery vs. no surgery (Supplementary Figure S1D) and surgery without neoadjuvant therapy vs. no surgery (Supplementary Figure S1E) in the T4N0-3M0 cohort, surgery vs. no surgery in stage IIIB-IVA adenocarcinoma (Supplementary Figure S4A) and squamous cell carcinoma (Supplementary Figure S4B) were all well balanced after PSM with all SMDs smaller than or very close to 0.1. All the results of Kaplan–Meier curves before and after PSM, multivariable Cox analysis, and PSM-adjusted Cox analysis indicated surgery brought better OS than no surgery in T4aN0-3M0 and T4bN0-3M0 cohort with all point estimates of HR < 0.5 and *P* < 0.001 (Fig. 1C–F, Table 3, and Supplementary Table S2–S3). Surgery also showed better OS than no surgery both in the settings of receiving and not receiving neoadjuvant therapy with both point estimates of HR < 0.6 and *P* < 0.001 in multivariable and PSM-adjusted Cox analysis (Fig. 3A–D, Table 3, and Supplementary Table S4–S5). In addition, whether neoadjuvant therapy could bring OS benefit was explored in patients who underwent surgery. Although the neoadjuvant therapy plus surgery group showed better OS than surgery without neoadjuvant therapy group in univariable analyses (Fig. 3E, Table 3, and Supplementary Table S6), no significant difference was found after adjusting the confounders using multivariable Cox analysis [0.966 (0.686–1.360) (*P* = 0.844)] or PSM-adjusted Cox analysis [0.892 (0.58–1.372) (*P* = 0.603)] (Fig. 3F, Table 3, and Supplementary Table S6). The surgery group showed better OS both in stage IIIB-IVA adenocarcinoma and squamous cell carcinoma (Supplementary Figure S4–5, Table 3, and Supplementary Table S7–S8).

## Discussion

The landmark RCT CROSS trial established the standard therapeutic strategy of neoadjuvant chemoradiotherapy plus surgery in resectable locally advanced esophageal cancer or esophagogastric junctional cancer [7]. However, no T4 esophageal cancer was included in the CROSS trial. Two options could be considered for this special population in clinical practice: neoadjuvant chemoradiotherapy plus surgery which was esophagectomy after downstaging of the cancer using chemoradiotherapy, and definitive chemoradiotherapy which was carried out with the maximum doses of irradiation [16, 17]. The role of surgery in T4N0-3M0 esophageal cancer was unclear for the time being, so we performed this population-based multicenter study and found surgery could decrease half of the all-cause death risk for this population. The subgroup analyses showed similar results. As the NCCN guideline for esophageal and esophagogastric junction cancers only recommend chemoradiotherapy or chemotherapy alone for T4b disease [4], sensitivity analysis in T4bN0-3M0 cohort was carried out and showed consistent results favoring surgery. In addition, surgery with and without neoadjuvant therapy could both achieve OS benefits in T4 disease. However, no significant difference in OS was observed between surgery with and without neoadjuvant therapy.

Only a few studies exploring the role of surgery in T4N0-3M0 esophageal cancer were reported. A prospective study (*n* = 53) conducted by Fujita et al. comparing esophagectomy after chemoradiotherapy and chemoradiotherapy alone in AJCC 6th T4N0-1M0 squamous cell carcinoma in thoracic esophagus suggested that surgery did not decrease the mortality risk for responders to chemoradiotherapy, but the nonresponders showed a tendency to benefit from esophagectomy without statistical significance [10]. Unlike this one, our study presented significant survival improvement in the surgery group for T4N0-3M0 esophageal cancer with HR close to 0.5 and *P* < 0.001. These different results might be caused by a different study cohort of only squamous cell carcinoma in the thoracic esophagus and a very small sample size in Hiromasa et al.’s study. Two retrospective studies directly compared surgery with no surgery in T4 esophageal cancer, reporting unfavorable results for surgery [12, 18]. Yamaguchi et al. performed a study consisting of 71 patients with esophageal cancer invading the trachea or bronchus and compared definitive chemoradiotherapy (*n* = 58) with induction chemoradiotherapy plus surgery (*n* = 13), and no significant OS difference was observed [12]. Makino et al.’s systemic review found induction therapy plus surgery was superior to definitive chemoradiotherapy regarding local disease control and short-term survival, however, the surgery group with higher perioperative mortality/morbidity, be noted, the long-term survival of the two groups could not be compared directly [18]. All these previous studies showed limited evidence because of the small sample size or bias brought by no adjustment for confounders. This suggest that our SEER database-based study had the merit of direct comparation between different treatment strategy adjusting the covariables with a big sample size.

In the present study, it is surprising to find that the neoadjuvant therapy did not prolong the OS time in the surgery group in the multivariable analyses, because patients with neoadjuvant therapy were more likely to have difficulty achieving an R0 resection which could cause worse prognosis. In a prospective study, Shimoji et al. compared the survival outcome of the unresectable T4 esophageal cancer undergoing induction chemotherapy/chemoradiotherapy with the initially resectable esophageal cancer undergoing esophagectomy immediately [14]. The R0 resection rate, in-hospital mortality rate, and 5-year OS rate of the induction group were significantly poorer than the no-induction group. However, for patients who achieved R0 resection, the induction group and no induction group showed no significant difference in the 5-year OS rate. All these results indicated that neoadjuvant therapy could bring opportunities for surgery and obtain satisfactory OS for unresectable T4 esophageal cancer if R0 resection could be achieved.

As shown in Table 1, only 18.9% (*n* = 344) of the whole study cohort (*n* = 1822) underwent surgery, indicating a small proportion of T4N0-3M0 esophageal cancers were resectable probably. For some unresectable T4 esophageal cancer patients, resectability could be achieved after chemoradiotherapy [19, 20]. Shimoji et al.’s study presented that surgery brought no significant difference in OS outcome for those unresectable tumors that responded to chemotherapy/chemoradiotherapy compared with initially resectable ones on the condition that R0 resection was achieved [14]. Therefore, the evaluation of the feasibility of complete resection is of large importance, as Shimada et al. also found that the most important prognostic factor for T4 esophageal cancer was the degree of curability of resection [21]. Taken together, comorbidities, performance status, the extent of invasion of the tumor, possibility of R0 resection, tolerance and response to chemoradiotherapy, new therapeutic techniques or new drugs like immune checkpoint inhibitors are all important factors influencing the extent of OS improvement brought by surgery. Clinicopathologic characteristics of T4 esophageal cancer patients are complicated and heterogeneous, so surgeons, oncologists, radiologists, and translational researchers should work together as a multiple-disciplinary team to make an optimal decision for this special population. What’s more, artificial intelligence models fed with live-streaming electronic health record data can be used to help doctors in decision-making perioperatively [22, 23].

Some limitations of the present study have to be admitted. First, there may be some bias that cannot be adjusted because of the study’s retrospective nature. Second, a Mixture of no and unknown radiotherapy/chemotherapy in the SEER database, and some factors like exposure to smoking/alcohol, multiple advanced underlying diseases performance status, regimens of chemotherapy, dose of radiation, R0/1/2 resection can influence the survival outcome but were not recorded in SEER database. Hence, RCTs with a large sample size are needed to validate the benefit of surgery.

Surgery might bring OS improvement for T4N0-3M0 esophageal cancer patients, no matter in T4a disease or T4b disease. Surgery with and without neoadjuvant therapy might both achieve better OS than no surgery.

### Supplementary Information

**Additional file 1: Supplementary Table S1.** Selection procedure of study cohort.** Supplementary Table S2.** Univariable and multivariable Cox regression analysis comparing no surgery with surgery for the OS of AJCC 8th T4aN0-3M0 EC patients. **Supplementary Table S3.** Univariable and multivariable Cox regression analysis comparing no surgery with surgery for the OS of AJCC 8th T4bN0-3M0 EC patients. **Supplementary Table S4.** Univariable and multivariable Cox regression analysis comparing no surgery with neoadjuvant therapy plus surgery for the OS of AJCC 8th T4N0-3M0 EC patients. **Supplementary Table S5.** Univariable and multivariable Cox regression analysis comparing no surgery with no neoadjuvant therapy plus surgery for the OS of AJCC 8th T4N0-3M0 EC patients. **Supplementary Table S6.** Univariable and multivariable Cox regression analysis comparing neoadjuvant therapy plus surgery with no neoadjuvant therapy plus surgery for the OS of AJCC 8th T4N0-3M0 EC patients. **Supplementary Table S7.** Univariable and multivariable Cox regression analysis comparing no surgery with surgery for the OS of AJCC 8th IIIB-IVA esophageal adenocarcinoma patients. **Supplementary Table S8.** Univariable and multivariable Cox regression analysis comparing no surgery with surgery for the OS of AJCC 8th IIIB-IVA esophageal squamous cell carcinoma patients. **Supplementary Figure S1.** Baseline standardized mean difference before and after PSM for surgery vs. no surgery in T4N0-3M0 EC (A), surgery vs. no surgery in T4aN0-3M0 EC (B), surgery vs. no surgery in T4bN0-3M0 EC (C), Neo + surgery vs. no surgery in T4N0-3M0 EC (D), surgery without Neo vs. no surgery in T4N0-3M0 EC (E), and Neo + surgery vs. surgery without Neo in T4N0-3M0 EC (F). PSM, propensity score matching; EC, esophageal cancer; Neo, neoadjuvant therapy. **Supplementary Figure S2.** Subgroup analysis of HR for surgery vs. no surgery in OS of stage T4aN0-3M0 EC. HR, hazard ratio; OS, overall survival; EC, esophageal cancer; CI, confidential interval. **Supplementary Figure S3.** Subgroup analysis of HR for surgery vs. no surgery in OS of stage T4bN0-3M0 EC. HR, hazard ratio; OS, overall survival; EC, esophageal cancer; CI, confidential interval. **Supplementary Figure S4.** Baseline standardized mean difference before and after PSM for surgery vs. no surgery in IIIB-IVA esophageal adenocarcinoma patients (A), and IIIB-IVA esophageal squamous cell carcinoma patients (B). PSM, propensity score matching. **Supplementary Figure S5.** Survival curves of OS for stage IIIB-IVA esophageal adenocarcinoma comparing surgery with no surgery before PSM (A) and after PSM (B), IIIB-IVA esophageal squamous cell carcinoma comparing surgery with no surgery before PSM (C) and after PSM (D). OS, overall survival; PSM, propensity score matching.

## Data Availability

All data used in the study can be downloaded from SEER*Stat software version 8.4.0 (https://seer.cancer.gov/).

## Abbreviations

AJCC: American Joint Committee on Cancer
CI: Confidence interval
HR: Hazard ratio
ICD-O-3: International Classification of Disease for Oncology
NCCN: National Comprehensive Cancer Network
OS: Overall survival
PSM: Propensity score matching
RCT: Randomized controlled trial
SEER: Surveillance, Epidemiology, and End Results
SMD: Standardized mean difference

## References

[1] Sung H, Ferlay J, Siegel RL, Laversanne M, Soerjomataram I, Jemal A, Bray F (2021). Global Cancer Statistics 2020: GLOBOCAN estimates of incidence and mortality worldwide for 36 cancers in 185 countries. CA Cancer J Clin.

[2] Rice TW, Patil DT, Blackstone EH (2017). 8th edition AJCC/UICC staging of cancers of the esophagus and esophagogastric junction: application to clinical practice. Ann Cardiothorac Surg.

[3] Gamliel Z, Krasna MJ (2005). Multimodality treatment of esophageal cancer. Surg Clin North Am.

[4] NCCN Guidelines Version 3.2023: Esophageal and Esophagogastric Junction Cancers [https://www.nccn.org/guidelines/guidelines-detail?category=1&id=1433]

[5] Takakusagi Y, Kano K, Shima S, Tsuchida K, Mizoguchi N, Katoh H, Kamada T, Taniuchi R, Iino M, Aoshika T (2022). Clinical outcomes of radiotherapy in elderly and younger patients with T4 esophageal cancer: a retrospective single-center analysis. Anticancer Res.

[6] Cooper JS, Guo MD, Herskovic A, Macdonald JS, Martenson JA, Al-Sarraf M, Byhardt R, Russell AH, Beitler JJ, Spencer S (1999). Chemoradiotherapy of locally advanced esophageal cancer: long-term follow-up of a prospective randomized trial (RTOG 85–01). Radiat Ther Oncol Group JAMA.

[7] Shapiro J, van Lanschot JJB, Hulshof M, van Hagen P, van Berge Henegouwen MI, Wijnhoven BPL, van Laarhoven HWM, Nieuwenhuijzen GAP, Hospers GAP, Bonenkamp JJ (2015). Neoadjuvant chemoradiotherapy plus surgery versus surgery alone for oesophageal or junctional cancer (CROSS): long-term results of a randomised controlled trial. Lancet Oncol.

[8] Makino T, Yamasaki M, Tanaka K, Tatsumi M, Takiguchi S, Hatazawa J, Mori M, Doki Y (2017). Importance of positron emission tomography for assessing the response of primary and metastatic lesions to induction treatments in T4 esophageal cancer. Surgery.

[9] Ohtsu A, Boku N, Muro K, Chin K, Muto M, Yoshida S, Satake M, Ishikura S, Ogino T, Miyata Y (1999). Definitive chemoradiotherapy for T4 and/or M1 lymph node squamous cell carcinoma of the esophagus. J Clin Oncol.

[10] Fujita H, Sueyoshi S, Tanaka T, Tanaka Y, Matono S, Mori N, Shirouzu K, Yamana H, Suzuki G, Hayabuchi N, Matsui M (2005). Esophagectomy: is it necessary after chemoradiotherapy for a locally advanced T4 esophageal cancer? Prospective nonrandomized trial comparing chemoradiotherapy with surgery versus without surgery. World J Surg.

[11] Satake H, Tahara M, Mochizuki S, Kato K, Hara H, Yokota T, Kiyota N, Kii T, Chin K, Zenda S (2016). A prospective, multicenter phase I/II study of induction chemotherapy with docetaxel, cisplatin and fluorouracil (DCF) followed by chemoradiotherapy in patients with unresectable locally advanced esophageal carcinoma. Cancer Chemother Pharmacol.

[12] Yamaguchi S, Morita M, Yamamoto M, Egashira A, Kawano H, Kinjo N, Tsujita E, Minami K, Ikebe M, Ikeda Y (2018). Long-term outcome of definitive chemoradiotherapy and induction chemoradiotherapy followed by surgery for T4 esophageal cancer with tracheobronchial invasion. Ann Surg Oncol.

[13] Gao LR, Li C, Han W, Ni W, Deng W, Tan L, Zhou Z, Chen D, Feng Q, Liang J (2022). Survival benefit of surgery in patients with clinical T4 esophageal cancer who achieved complete or partial response after neoadjuvant chemoradiotherapy or radiotherapy. Ther Adv Med Oncol.

[14] Shimoji H, Karimata H, Nagahama M, Nishimaki T (2013). Induction chemotherapy or chemoradiotherapy followed by radical esophagectomy for T4 esophageal cancer: results of a prospective cohort study. World J Surg.

[15] He H, Chen N, Hou Y, Wang Z, Zhang Y, Zhang G, Fu J (2020). Trends in the incidence and survival of patients with esophageal cancer: a SEER database analysis. Thorac Cancer.

[16] Ji Y, Du X, Zhu W, Yang Y, Ma J, Zhang L, Li J, Tao H, Xia J, Yang H (2021). Efficacy of concurrent chemoradiotherapy with s-1 vs radiotherapy alone for older patients with esophageal cancer: a multicenter randomized phase 3 clinical trial. JAMA Oncol.

[17] Kamarajah SK, Phillips AW, Hanna GB, Low D, Markar SR (2022). Definitive chemoradiotherapy compared to neoadjuvant chemoradiotherapy with esophagectomy for locoregional esophageal cancer: national population-based cohort study. Ann Surg.

[18] Makino T, Yamasaki M, Tanaka K, Miyazaki Y, Takahashi T, Kurokawa Y, Motoori M, Kimura Y, Nakajima K, Mori M, Doki Y (2019). Treatment and clinical outcome of clinical T4 esophageal cancer: a systematic review. Ann Gastroenterol Surg.

[19] Akutsu Y, Kono T, Uesato M, Hoshino I, Murakami K, Aoyagi T, Ota T, Toyozumi T, Suito H, Kobayashi H (2014). Is the outcome of a salvage surgery for T4 thoracic esophageal squamous cell carcinoma really poor?. World J Surg.

[20] Akutsu Y, Matsubara H (2015). Chemoradiotherapy and surgery for T4 esophageal cancer in Japan. Surg Today.

[21] Shimada H, Shiratori T, Okazumi S, Matsubara H, Nabeya Y, Shuto K, Akutsu Y, Hayashi H, Isono K, Ochiai T (2008). Have surgical outcomes of pathologic T4 esophageal squamous cell carcinoma really improved? Analysis of 268 cases during 45 years of experience. J Am Coll Surg.

[22] Loftus TJ, Tighe PJ, Filiberto AC, Efron PA, Brakenridge SC, Mohr AM, Rashidi P, Upchurch GR, Bihorac A (2020). Artificial intelligence and surgical decision-making. JAMA Surg.

[23] Chadebecq F, Lovat LB, Stoyanov D (2023). Artificial intelligence and automation in endoscopy and surgery. Nat Rev Gastroenterol Hepatol.

