# Supplementary material for: Role of surgery in T4N0-3M0 esophageal cancer
Source: World J Surg Oncol. 2023 Nov 27;21:369. doi: 10.1186/s12957-023-03239-8 (PMC10680323; doi:10.1186/s12957-023-03239-8)
Supplement: Supplementary file 1 — Additional file 1: Supplementary Table S1. Selection procedure of study cohort. Supplementary Table S2. Univariable and multivariable Cox regression analysis comparing no surgery with surgery for the OS of AJCC 8th T4aN0-3M0 EC patients. Supplementary Table S3. Univariable and multivariable Cox regression analysis comparing no surgery with surgery for the OS of AJCC 8th T4bN0-3M0 EC patients. Supplementary Table S4. Univariable and multivariable Cox regression analysis comparing no surgery with neoadjuvant therapy plus surgery for the OS of AJCC 8th T4N0-3M0 EC patients. Supplementary Table S5. Univariable and multivariable Cox regression analysis comparing no surgery with no neoadjuvant therapy plus surgery for the OS of AJCC 8th T4N0-3M0 EC patients. Supplementary Table S6. Univariable and multivariable Cox regression analysis comparing neoadjuvant therapy plus surgery with no neoadjuvant therapy plus surgery for the OS of AJCC 8th T4N0-3M0 EC patients. Supplementary Table S7. Univariable and multivariable Cox regression analysis comparing no surgery with surgery for the OS of AJCC 8th IIIB-IVA esophageal adenocarcinoma patients. Supplementary Table S8. Univariable and multivariable Cox regression analysis comparing no surgery with surgery for the OS of AJCC 8th IIIB-IVA esophageal squamous cell carcinoma patients. Supplementary Figure S1. Baseline standardized mean difference before and after PSM for surgery vs. no surgery in T4N0-3M0 EC (A), surgery vs. no surgery in T4aN0-3M0 EC (B), surgery vs. no surgery in T4bN0-3M0 EC (C), Neo + surgery vs. no surgery in T4N0-3M0 EC (D), surgery without Neo vs. no surgery in T4N0-3M0 EC (E), and Neo + surgery vs. surgery without Neo in T4N0-3M0 EC (F). PSM, propensity score matching; EC, esophageal cancer; Neo, neoadjuvant therapy. Supplementary Figure S2. Subgroup analysis of HR for surgery vs. no surgery in OS of stage T4aN0-3M0 EC. HR, hazard ratio; OS, overall survival; EC, esophageal cancer; CI, confidential interval. [file 12957_2023_3239_MOESM1_ESM.docx]

**Supplementary material**

**Supplementary Table 1** Selection procedure of study cohort

**Supplementary Table 2** Univariable and multivariable Cox regression analysis comparing no surgery with surgery for the OS of AJCC 8th T4aN0-3M0 EC patients

**Supplementary Table 3** Univariable and multivariable Cox regression analysis comparing no surgery with surgery for the OS of AJCC 8th T4bN0-3M0 EC patients

**Supplementary Table 4** Univariable and multivariable Cox regression analysis comparing no surgery with neoadjuvant therapy plus surgery for the OS of AJCC 8th T4N0-3M0 EC patients

**Supplementary Table 5** Univariable and multivariable Cox regression analysis comparing no surgery with no neoadjuvant therapy plus surgery for the OS of AJCC 8th T4N0-3M0 EC patients

**Supplementary Table 6** Univariable and multivariable Cox regression analysis comparing neoadjuvant therapy plus surgery with no neoadjuvant therapy plus surgery for the OS of AJCC 8th T4N0-3M0 EC patients

**Supplementary Table 7** Univariable and multivariable Cox regression analysis comparing no surgery with surgery for the OS of AJCC 8th IIIB-IVA esophageal adenocarcinoma patients

**Supplementary Table 8** Univariable and multivariable Cox regression analysis comparing no surgery with surgery for the OS of AJCC 8th IIIB-IVA esophageal squamous cell carcinoma patients

**Supplementary Figure 1** Baseline standardized mean difference before and after PSM for surgery vs. no surgery in T4N0-3M0 EC (A), surgery vs. no surgery in T4aN0-3M0 EC (B), surgery vs. no surgery in T4bN0-3M0 EC (C), Neo + surgery vs. no surgery in T4N0-3M0 EC (D), surgery without Neo vs. no surgery in T4N0-3M0 EC (E), and Neo + surgery vs. surgery without Neo in T4N0-3M0 EC (F). PSM, propensity score matching; EC, esophageal cancer; Neo, neoadjuvant therapy.

**Supplementary Figure 2** Subgroup analysis of HR for surgery vs. no surgery in OS of stage T4aN0-3M0 EC. HR, hazard ratio; OS, overall survival; EC, esophageal cancer; CI, confidential interval.

**Supplementary Figure 3** Subgroup analysis of HR for surgery vs. no surgery in OS of stage T4bN0-3M0 EC. HR, hazard ratio; OS, overall survival; EC, esophageal cancer; CI, confidential interval.

**Supplementary Figure 4** Baseline standardized mean difference before and after PSM for surgery vs. no surgery in IIIB-IVA esophageal adenocarcinoma patients (A), and IIIB-IVA esophageal squamous cell carcinoma patients (B). PSM, propensity score matching.

**Supplementary Figure 5** Survival curves of OS for stage IIIB-IVA esophageal adenocarcinoma comparing surgery with no surgery before PSM (A) and after PSM (B), IIIB-IVA esophageal squamous cell carcinoma comparing surgery with no surgery before PSM (C) and after PSM (D). OS, overall survival; PSM, propensity score matching.

**Supplementary Table 1 Selection procedure of study cohort**

| **Step** | **Criteria** | **Number excluded** | **Number remained** |
| --- | --- | --- | --- |
| 1 | Patients with 8th AJCC T4N0-3M0 (only one primary) EC between 2004 and 2015 |  | 1826 |
| 2 | Exclude if aged <18 years | 0 | 1826 |
| 3 | Exclude if diagnosed with autopsy/death certificate only or diagnosed without pathological confirmation | 0 | 1826 |
| 4 | Exclude if whether surgery was performed was unknown | 4 | 1822 |

EC, esophageal cancer.

**Supplementary Table 2** Univariable and multivariable Cox regression analysis comparing no surgery with surgery for the OS of AJCC 8th T4aN0-3M0 EC patients

| **Variables** | **Univariable analysis** | | **Multivariable analysis** | |  |
| --- | --- | --- | --- | --- | --- |
|  | **HR (95%CI)** | **P** | **HR (95%CI)** | **P** |  |
| **Surgery** |  |  |  |  |  |
| No | 1 |  | 1 |  |  |
| Yes | 0.377 (0.289-0.493) | <0.001 | 0.471 (0.354-0.627) | <0.001 |  |
| **Year of diagnosis** |  |  |  |  |  |
| 2004-2009 | 1 |  | 1 |  |  |
| 2010-2015 | 0.740 (0.534-1.026) | 0.071 | 0.999 (0.705-1.417) | 0.997 |  |
| **Age** |  |  |  |  |  |
| <65 years old | 1 |  | 1 |  |  |
| >=65 years old | 1.320 (1.073-1.624) | 0.009 | 1.151 (0.924-1.434) | 0.21 |  |
| **Sex** |  |  |  |  |  |
| Male | 1 |  |  |  |  |
| Female | 1.049 (0.815-1.349) | 0.711 |  |  |  |
| **Race** |  |  |  |  |  |
| White | 1 |  | 1 |  |  |
| Black | 1.471 (1.074-2.016) | 0.016 | 1.236 (0.854-1.790) | 0.261 |  |
| Other/Unknown | 0.849 (0.560-1.286) | 0.439 | 0.790 (0.513-1.218) | 0.286 |  |
| **Primary site** |  |  |  |  |  |
| Upper third | 1 |  | 1 |  |  |
| Middle third | 1.324 (0.841-2.085) | 0.225 | 1.358 (0.832-2.219) | 0.221 |  |
| Lower third | 1.001 (0.707-1.417) | 0.996 | 1.014 (0.641-1.604) | 0.953 |  |
| Unknown | 1.449 (0.956-2.198) | 0.081 | 1.106 (0.679-1.804) | 0.685 |  |
| **Histology** |  |  |  |  |  |
| Adenocarcinoma | 1 |  | 1 |  |  |
| Squamous cell carcinoma | 1.225 (0.976-1.538) | 0.08 | 1.159 (0.821-1.635) | 0.402 |  |
| Other | 1.603 (1.087-2.364) | 0.017 | 1.443 (0.967-2.154) | 0.073 |  |
| **Differentiation** |  |  |  |  |  |
| Grade I | 1 |  |  |  |  |
| Grade II | 0.969 (0.555-1.693) | 0.912 |  |  |  |
| Grade III/IV | 1.190 (0.690-2.052) | 0.532 |  |  |  |
| Unknown | 1.071 (0.604-1.899) | 0.814 |  |  |  |
| **N stage** |  |  |  |  |  |
| N0 | 1 |  |  |  |  |
| N1 | 0.908 (0.723-1.142) | 0.41 |  |  |  |
| N2 | 0.762 (0.524-1.108) | 0.155 |  |  |  |
| N3 | 1.230 (0.743-2.037) | 0.422 |  |  |  |
| N1-3, NOS | 1.208 (0.739-1.974) | 0.451 |  |  |  |
| **Radiotherapy** |  |  |  |  |  |
| No/Unknown | 1 |  | 1 |  |  |
| Yes | 0.396 (0.317-0.494) | <0.001 | 0.514 (0.388-0.681) | <0.001 |  |
| **Chemotherapy** |  |  |  |  |  |
| No/Unknown | 1 |  | 1 |  |  |
| Yes | 0.299 (0.238-0.377) | <0.001 | 0.472 (0.354-0.629) | <0.001 |  |

OS, overall survival; EC, esophageal cancer; HR, hazard ratio; CI, confidential interval; NOS, not otherwise specified.

**Supplementary Table 3** Univariable and multivariable Cox regression analysis comparing no surgery with surgery for the OS of AJCC 8th T4bN0-3M0 EC patients

| **Variables** | **Univariable analysis** | | **Multivariable analysis** | |  |
| --- | --- | --- | --- | --- | --- |
|  | **HR (95%CI)** | **P** | **HR (95%CI)** | **P** |  |
| **Surgery** |  |  |  |  |  |
| No | 1 |  | 1 |  |  |
| Yes | 0.423 (0.297-0.603) | <0.001 | 0.480 (0.335-0.689) | <0.001 |  |
| **Year of diagnosis** |  |  |  |  |  |
| 2004-2009 | 1 |  |  |  |  |
| 2010-2015 | 0.904 (0.726-1.126) | 0.369 |  |  |  |
| **Age** |  |  |  |  |  |
| <65 years old | 1 |  | 1 |  |  |
| >=65 years old | 1.180 (0.982-1.419) | 0.078 | 1.094 (0.907-1.320) | 0.346 |  |
| **Sex** |  |  |  |  |  |
| Male | 1 |  |  |  |  |
| Female | 1.012 (0.823-1.245) | 0.908 |  |  |  |
| **Race** |  |  |  |  |  |
| White | 1 |  |  |  |  |
| Black | 1.193 (0.960-1.482) | 0.112 |  |  |  |
| Other/Unknown | 0.940 (0.695-1.273) | 0.691 |  |  |  |
| **Primary site** |  |  |  |  |  |
| Upper third | 1 |  | 1 |  |  |
| Middle third | 1.041 (0.798-1.358) | 0.767 | 1.059 (0.807-1.389) | 0.679 |  |
| Lower third | 0.920 (0.710-1.194) | 0.532 | 1.042 (0.773-1.404) | 0.789 |  |
| Unknown | 1.321 (1.006-1.736) | 0.045 | 1.148 (0.866-1.523) | 0.337 |  |
| **Histology** |  |  |  |  |  |
| Adenocarcinoma | 1 |  | 1 |  |  |
| Squamous cell carcinoma | 1.320 (1.053-1.656) | 0.016 | 1.168 (0.894-1.527) | 0.256 |  |
| Other | 1.590 (1.125-2.247) | 0.009 | 1.081 (0.742-1.574) | 0.684 |  |
| **Differentiation** |  |  |  |  |  |
| Grade I | 1 |  | 1 |  |  |
| Grade II | 1.445 (0.875-2.386) | 0.151 | 1.473 (0.884-2.456) | 0.137 |  |
| Grade III/IV | 1.689 (1.026-2.781) | 0.039 | 1.764 (1.062-2.930) | 0.028 |  |
| Unknown | 1.726 (1.031-2.890) | 0.038 | 1.527 (0.899-2.592) | 0.117 |  |
| **N stage** |  |  |  |  |  |
| N0 | 1 |  |  |  |  |
| N1 | 0.844 (0.684-1.042) | 0.116 |  |  |  |
| N2 | 0.923 (0.667-1.278) | 0.631 |  |  |  |
| N3 | 1.102 (0.685-1.773) | 0.688 |  |  |  |
| N1-3, NOS | 1.039 (0.742-1.454) | 0.825 |  |  |  |
| **Radiotherapy** |  |  |  |  |  |
| No/Unknown | 1 |  | 1 |  |  |
| Yes | 0.388 (0.318-0.472) | <0.001 | 0.541 (0.433-0.676) | <0.001 |  |
| **Chemotherapy** |  |  |  |  |  |
| No/Unknown | 1 |  | 1 |  |  |
| Yes | 0.362 (0.298-0.439) | <0.001 | 0.474 (0.379-0.592) | <0.001 |  |

OS, overall survival; EC, esophageal cancer; HR, hazard ratio; CI, confidential interval; NOS, not otherwise specified.

**Supplementary Table 4** Univariable and multivariable Cox regression analysis comparing no surgery with neoadjuvant therapy plus surgery for the OS of AJCC 8th T4N0-3M0 EC patients

| **Variables** | **Univariable analysis** | | **Multivariable analysis** | |  |
| --- | --- | --- | --- | --- | --- |
|  | **HR (95%CI)** | **P** | **HR (95%CI)** | **P** |  |
| **Surgery** |  |  |  |  |  |
| No | 1 |  | 1 |  |  |
| Neo + surgery | 0.373 (0.319-0.437) | <0.001 | 0.548 (0.461-0.650) | <0.001 |  |
| **Year of diagnosis** |  |  |  |  |  |
| 2004-2009 | 1 |  |  |  |  |
| 2010-2015 | 0.940 (0.851-1.038) | 0.222 |  |  |  |
| **Age** |  |  |  |  |  |
| <65 years old | 1 |  | 1 |  |  |
| >=65 years old | 1.238 (1.122-1.366) | 0.002 | 1.102 (0.995-1.221) | 0.061 |  |
| **Sex** |  |  |  |  |  |
| Male | 1 |  |  |  |  |
| Female | 1.043 (0.929-1.171) | 0.477 |  |  |  |
| **Race** |  |  |  |  |  |
| White | 1 |  | 1 |  |  |
| Black | 1.390 (1.225-1.577) | <0.001 | 1.068 (0.928-1.228) | 0.359 |  |
| Other/Unknown | 0.923 (0.763-1.116) | 0.41 | 0.828 (0.682-1.006) | 0.058 |  |
| **Primary site** |  |  |  |  |  |
| Upper third | 1 |  | 1 |  |  |
| Middle third | 1.180 (1.002-1.389) | 0.047 | 1.235 (1.044-1.460) | 0.014 |  |
| Lower third | 0.859 (0.746-0.989) | 0.034 | 1.078 (0.911-1.277) | 0.382 |  |
| Unknown | 1.287 (1.091-1.518) | 0.003 | 1.191 (1.003-1.414) | 0.046 |  |
| **Histology** |  |  |  |  |  |
| Adenocarcinoma | 1 |  | 1 |  |  |
| Squamous cell carcinoma | 1.386 (1.250-1.537) | <0.001 | 1.322 (1.149-1.522) | <0.001 |  |
| Other | 1.619 (1.335-1.963) | <0.001 | 1.324 (1.081-1.620) | 0.007 |  |
| **Differentiation** |  |  |  |  |  |
| Grade I | 1 |  | 1 |  |  |
| Grade II | 1.144 (0.872-1.500) | 0.331 | 1.111 (0.846-1.460) | 0.449 |  |
| Grade III/IV | 1.339 (1.024-1.752) | 0.033 | 1.356 (1.035-1.777) | 0.027 |  |
| Unknown | 1.246 (0.942-1.648) | 0.123 | 1.093 (0.824-1.449) | 0.538 |  |
| **T stage** |  |  |  |  |  |
| T4a | 1 |  | 1 |  |  |
| T4b | 1.566 (1.361-1.802) | <0.001 | 1.220 (1.051-1.416) | 0.009 |  |
| T4, NOS | 1.247 (1.099-1.414) | 0.001 | 1.101 (0.955-1.270) | 0.184 |  |
| **N stage** |  |  |  |  |  |
| N0 | 1 |  | 1 |  |  |
| N1 | 0.834 (0.736-0.944) | 0.004 | 1.065 (0.931-1.218) | 0.361 |  |
| N2 | 0.804 (0.646-0.999) | 0.049 | 1.034 (0.825-1.297) | 0.77 |  |
| N3 | 1.137 (0.832-1.553) | 0.421 | 1.611 (1.169-2.221) | 0.004 |  |
| N1-3, NOS | 1.036 (0.914-1.175) | 0.576 | 1.140 (1.000-1.301) | 0.05 |  |
| **Radiotherapy** |  |  |  |  |  |
| No/Unknown | 1 |  | 1 |  |  |
| Yes | 0.388 (0.349-0.432) | <0.001 | 0.555 (0.490-0.629) | <0.001 |  |
| **Chemotherapy** |  |  |  |  |  |
| No/Unknown | 1 |  | 1 |  |  |
| Yes | 0.321 (0.288-0.357) | <0.001 | 0.473 (0.416-0.538) | <0.001 |  |

OS, overall survival; EC, esophageal cancer; HR, hazard ratio; CI, confidential interval; Neo, neoadjuvant therapy; NOS, not otherwise specified.

**Supplementary Table 5** Univariable and multivariable Cox regression analysis comparing no surgery with no neoadjuvant therapy plus surgery for the OS of AJCC 8th T4N0-3M0 EC patients

| **Variables** | **Univariable analysis** | | **Multivariable analysis** | |  |
| --- | --- | --- | --- | --- | --- |
|  | **HR (95%CI)** | **P** | **HR (95%CI)** | **P** |  |
| **Surgery** |  |  |  |  |  |
| No | 1 |  | 1 |  |  |
| Surgery without Neo | 0.628 (0.512-0.769) | <0.001 | 0.464 (0.375-0.574) | <0.001 |  |
| **Year of diagnosis** |  |  |  |  |  |
| 2004-2009 | 1 |  |  |  |  |
| 2010-2015 | 1.021 (0.922-1.131) | 0.686 |  |  |  |
| **Age** |  |  |  |  |  |
| <65 years old | 1 |  | 1 |  |  |
| >=65 years old | 1.204 (1.088-1.333) | <0.001 | 1.117 (1.005-1.241) | 0.039 |  |
| **Sex** |  |  |  |  |  |
| Male | 1 |  |  |  |  |
| Female | 0.981 (0.872-1.103) | 0.745 |  |  |  |
| **Race** |  |  |  |  |  |
| White | 1 |  | 1 |  |  |
| Black | 1.196 (1.055-1.356) | 0.005 | 1.043 (0.908-1.198) | 0.548 |  |
| Other/Unknown | 0.939 (0.771-1.143) | 0.531 | 0.849 (0.694-1.039) | 0.112 |  |
| **Primary site** |  |  |  |  |  |
| Upper third | 1 |  | 1 |  |  |
| Middle third | 1.341 (1.139-1.579) | <0.001 | 1.303 (1.104-1.538) | 0.002 |  |
| Lower third | 1.041 (0.903-1.200) | 0.583 | 1.078 (0.911-1.277) | 0.381 |  |
| Unknown | 1.361 (1.154-1.605) | <0.001 | 1.187 (0.999-1.409) | 0.051 |  |
| **Histology** |  |  |  |  |  |
| Adenocarcinoma | 1 |  | 1 |  |  |
| Squamous cell carcinoma | 1.211 (1.088-1.348) | <0.001 | 1.340 (1.162-1.546) | <0.001 |  |
| Other | 1.467 (1.206-1.785) | <0.001 | 1.374 (1.120-1.686) | 0.002 |  |
| **Differentiation** |  |  |  |  |  |
| Grade I | 1 |  | 1 |  |  |
| Grade II | 1.203 (0.914-1.582) | 0.187 | 1.213 (0.920-1.601) | 0.171 |  |
| Grade III/IV | 1.388 (1.058-1.820) | 0.018 | 1.498 (1.138-1.972) | 0.004 |  |
| Unknown | 1.343 (1.012-1.782) | 0.041 | 1.234 (0.926-1.645) | 0.152 |  |
| **T stage** |  |  |  |  |  |
| T4a | 1 |  | 1 |  |  |
| T4b | 1.341 (1.158-1.552) | <0.001 | 1.186 (1.017-1.384) | 0.03 |  |
| T4, NOS | 1.127 (0.987-1.286) | 0.077 | 1.104 (0.964-1.264) | 0.151 |  |
| **N stage** |  |  |  |  |  |
| N0 | 1 |  |  |  |  |
| N1 | 0.935 (0.822-1.065) | 0.312 |  |  |  |
| N2 | 0.826 (0.657-1.039) | 0.103 |  |  |  |
| N3 | 1.117 (0.836-1.493) | 0.455 |  |  |  |
| N1-3, NOS | 1.044 (0.919-1.187) | 0.507 |  |  |  |
| **Radiotherapy** |  |  |  |  |  |
| No/Unknown | 1 |  | 1 |  |  |
| Yes | 0.469 (0.422-0.521) | <0.001 | 0.550 (0.485-0.623) | <0.001 |  |
| **Chemotherapy** |  |  |  |  |  |
| No/Unknown | 1 |  | 1 |  |  |
| Yes | 0.432 (0.389-0.480) | <0.001 | 0.520 (0.459-0.590) | <0.001 |  |

OS, overall survival; EC, esophageal cancer; HR, hazard ratio; CI, confidential interval; Neo, neoadjuvant therapy; NOS, not otherwise specified.

**Supplementary Table 6** Univariable and multivariable Cox regression analysis comparing neoadjuvant therapy plus surgery with no neoadjuvant therapy plus surgery for the OS of AJCC 8th T4N0-3M0 EC patients

| **Variables** | **Univariable analysis** | | **Multivariable analysis** | |  |
| --- | --- | --- | --- | --- | --- |
|  | **HR (95%CI)** | **P** | **HR (95%CI)** | **P** |  |
| **Neoadjuvant therapy** |  |  |  |  |  |
| No | 1 |  | 1 |  |  |
| Yes | 0.586 (0.459-0.748) | <0.001 | 0.966 (0.686-1.360) | 0.843 |  |
| **Year of diagnosis** |  |  |  |  |  |
| 2004-2009 | 1 |  | 1 |  |  |
| 2010-2015 | 0.704 (0.545-0.909) | 0.007 | 0.714 (0.438-1.163) | 0.176 |  |
| **Age** |  |  |  |  |  |
| <65 years old | 1 |  |  |  |  |
| >=65 years old | 1.102 (0.868-1.398) | 0.426 |  |  |  |
| **Sex** |  |  |  |  |  |
| Male | 1 |  |  |  |  |
| Female | 0.950 (0.690-1.307) | 0.751 |  |  |  |
| **Race** |  |  |  |  |  |
| White | 1 |  | 1 |  |  |
| Black | 1.540 (1.034-2.294) | 0.034 | 1.072 (0.681-1.687) | 0.765 |  |
| Other/Unknown | 0.665 (0.388-1.141) | 0.139 | 0.515 (0.295-0.901) | 0.02 |  |
| **Primary site** |  |  |  |  |  |
| Upper third | 1 |  |  |  |  |
| Middle third | 0.802 (0.461-1.396) | 0.435 |  |  |  |
| Lower third | 0.694 (0.433-1.113) | 0.129 |  |  |  |
| Unknown | 0.859 (0.491-1.503) | 0.595 |  |  |  |
| **Histology** |  |  |  |  |  |
| Adenocarcinoma | 1 |  | 1 |  |  |
| Squamous cell carcinoma | 1.457 (1.131-1.877) | 0.004 | 1.643 (1.223-2.208) | <0.001 |  |
| Other | 1.344 (0.661-2.732) | 0.414 | 1.417 (0.681-2.946) | 0.351 |  |
| **Differentiation** |  |  |  |  |  |
| Grade I | 1 |  |  |  |  |
| Grade II | 1.020 (0.610-1.706) | 0.94 |  |  |  |
| Grade III/IV | 1.237 (0.747-2.050) | 0.408 |  |  |  |
| Unknown | 0.729 (0.399-1.332) | 0.304 |  |  |  |
| **T stage** |  |  |  |  |  |
| T4a | 1 |  | 1 |  |  |
| T4b | 1.383 (0.917-2.086) | 0.122 | 1.214 (0.743-1.984) | 0.439 |  |
| T4, NOS | 1.371 (1.034-1.817) | 0.028 | 0.839 (0.502-1.403) | 0.503 |  |
| **N stage** |  |  |  |  |  |
| N0 | 1 |  | 1 |  |  |
| N1 | 1.107 (0.820-1.493) | 0.508 | 1.467 (1.058-2.032) | 0.021 |  |
| N2 | 1.685 (1.154-2.461) | 0.007 | 1.985 (1.337-2.947) | <0.001 |  |
| N3 | 2.483 (1.637-3.766) | <0.001 | 3.441 (2.195-5.393) | <0.001 |  |
| N1-3, NOS | 1.228 (0.834-1.806) | 0.298 | 1.463 (0.961-2.227) | 0.076 |  |
| **Regional nodes examined** |  |  |  |  |  |
| <15 | 1 |  | 1 |  |  |
| >=15 | 0.791 (0.618-1.014) | 0.064 | 0.658 (0.502-0.862) | 0.002 |  |
| Unknown | 0.000 (0.000-Inf) 0 | 0.992 | 0.000 (0.000-Inf) 0 | 0.993 |  |
| **Radiotherapy** |  |  |  |  |  |
| No/Unknown | 1 |  | 1 |  |  |
| Yes | 0.613 (0.465-0.806) | <0.001 | 0.731 (0.489-1.091) | 0.125 |  |
| **Chemotherapy** |  |  |  |  |  |
| No/Unknown | 1 |  | 1 |  |  |
| Yes | 0.498 (0.369-0.672) | <0.001 | 0.671 (0.413-1.090) | 0.107 |  |

OS, overall survival; EC, esophageal cancer; HR, hazard ratio; CI, confidential interval; NOS, not otherwise specified.

**Supplementary Table 7** Univariable and multivariable Cox regression analysis comparing no surgery with surgery for the OS of AJCC 8th IIIB-IVA esophageal adenocarcinoma patients

| **Variables** | **Univariable analysis** | | **Multivariable analysis** | |  |
| --- | --- | --- | --- | --- | --- |
|  | **HR (95%CI)** | **P** | **HR (95%CI)** | **P** |  |
| **Surgery** |  |  |  |  |  |
| No | 1 |  | 1 |  |  |
| Yes | 0.373 (0.289-0.481) | <0.001 | 0.432 (0.331-0.565) | <0.001 |  |
| **Year of diagnosis** |  |  |  |  |  |
| 2004-2009 | 1 |  | 1 |  |  |
| 2010-2015 | 0.686 (0.506-0.931) | 0.015 | 0.766 (0.549-1.069) | 0.117 |  |
| **Age** |  |  |  |  |  |
| <65 years old | 1 |  | 1 |  |  |
| >=65 years old | 1.313 (1.054-1.636) | 0.015 | 1.103 (0.878-1.384) | 0.401 |  |
| **Sex** |  |  |  |  |  |
| Male | 1 |  |  |  |  |
| Female | 1.182 (0.877-1.595) | 0.272 |  |  |  |
| **Race** |  |  |  |  |  |
| White | 1 |  | 1 |  |  |
| Black | 1.973 (1.048-3.716) | 0.035 | 1.633 (0.847-3.145) | 0.143 |  |
| Other/Unknown | 0.841 (0.515-1.371) | 0.486 | 0.707 (0.428-1.168) | 0.176 |  |
| **Primary site** |  |  |  |  |  |
| Upper third | 1 |  |  |  |  |
| Middle third | 0.679 (0.201-2.294) | 0.534 |  |  |  |
| Lower third | 0.73 (0.233-2.283) | 0.589 |  |  |  |
| Unknown | 1.025 (0.317-3.31) | 0.967 |  |  |  |
| **Differentiation** |  |  |  |  |  |
| Grade I | 1 |  |  |  |  |
| Grade II | 1.092 (0.584-2.043) | 0.782 |  |  |  |
| Grade III/IV | 1.494 (0.812-2.749) | 0.197 |  |  |  |
| Unknown | 1.21 (0.627-2.335) | 0.569 |  |  |  |
| **TNM stage** |  |  |  |  |  |
| IIIB | 1 |  | 1 |  |  |
| IVA | 1.349 (1.083-1.68) | 0.007 | 1.468 (1.156-1.864) | 0.002 |  |
| **Radiotherapy** |  |  |  |  |  |
| No/Unknown | 1 |  | 1 |  |  |
| Yes | 0.435 (0.346-0.547) | <0.001 | 0.651 (0.496-0.854) | 0.002 |  |
| **Chemotherapy** |  |  |  |  |  |
| No/Unknown | 1 |  | 1 |  |  |
| Yes | 0.311 (0.244-0.397) | <0.001 | 0.479 (0.354-0.649) | <0.001 |  |

OS, overall survival; HR, hazard ratio; CI, confidential interval.

**Supplementary Table 8** Univariable and multivariable Cox regression analysis comparing no surgery with surgery for the OS of AJCC 8th IIIB-IVA esophageal squamous cell carcinoma patients

| **Variables** | **Univariable analysis** | | **Multivariable analysis** | |  |
| --- | --- | --- | --- | --- | --- |
|  | **HR (95%CI)** | **P** | **HR (95%CI)** | **P** |  |
| **Surgery** |  |  |  |  |  |
| No | 1 |  | 1 |  |  |
| Yes | 0.511 (0.349-0.749) | 0.001 | 0.388 (0.26-0.579) | <0.001 |  |
| **Year of diagnosis** |  |  |  |  |  |
| 2004-2009 | 1 |  |  |  |  |
| 2010-2015 | 0.961 (0.702-1.316) | 0.805 |  |  |  |
| **Age** |  |  |  |  |  |
| <65 years old | 1 |  | 1 |  |  |
| >=65 years old | 1.212 (0.992-1.482) | 0.061 | 1.11 (0.906-1.36) | 0.314 |  |
| **Sex** |  |  |  |  |  |
| Male | 1 |  |  |  |  |
| Female | 0.854 (0.687-1.061) | 0.154 |  |  |  |
| **Race** |  |  |  |  |  |
| White | 1 |  |  |  |  |
| Black | 1.192 (0.957-1.485) | 0.116 |  |  |  |
| Other/Unknown | 0.833 (0.602-1.153) | 0.27 |  |  |  |
| **Primary site** |  |  |  |  |  |
| Upper third | 1 |  | 1 |  |  |
| Middle third | 1.252 (0.968-1.619) | 0.087 | 1.27 (0.98-1.647) | 0.071 |  |
| Lower third | 1.068 (0.783-1.458) | 0.677 | 1.052 (0.769-1.439) | 0.752 |  |
| Unknown | 1.436 (1.092-1.889) | 0.01 | 1.105 (0.835-1.462) | 0.484 |  |
| **Differentiation** |  |  |  |  |  |
| Grade I | 1 |  |  |  |  |
| Grade II | 1.175 (0.712-1.94) | 0.528 |  |  |  |
| Grade III/IV | 1.217 (0.733-2.021) | 0.448 |  |  |  |
| Unknown | 1.189 (0.708-1.995) | 0.513 |  |  |  |
| **TNM stage** |  |  |  |  |  |
| IIIB | 1 |  | 1 |  |  |
| IVA | 1.3 (1.025-1.648) | 0.03 | 1.043 (0.818-1.33) | 0.735 |  |
| **Radiotherapy** |  |  |  |  |  |
| No/Unknown | 1 |  | 1 |  |  |
| Yes | 0.315 (0.252-0.394) | <0.001 | 0.481 (0.371-0.623) | <0.001 |  |
| **Chemotherapy** |  |  |  |  |  |
| No/Unknown | 1 |  | 1 |  |  |
| Yes | 0.295 (0.238-0.367) | <0.001 | 0.356 (0.275-0.46) | <0.001 |  |

OS, overall survival; HR, hazard ratio; CI, confidential interval.


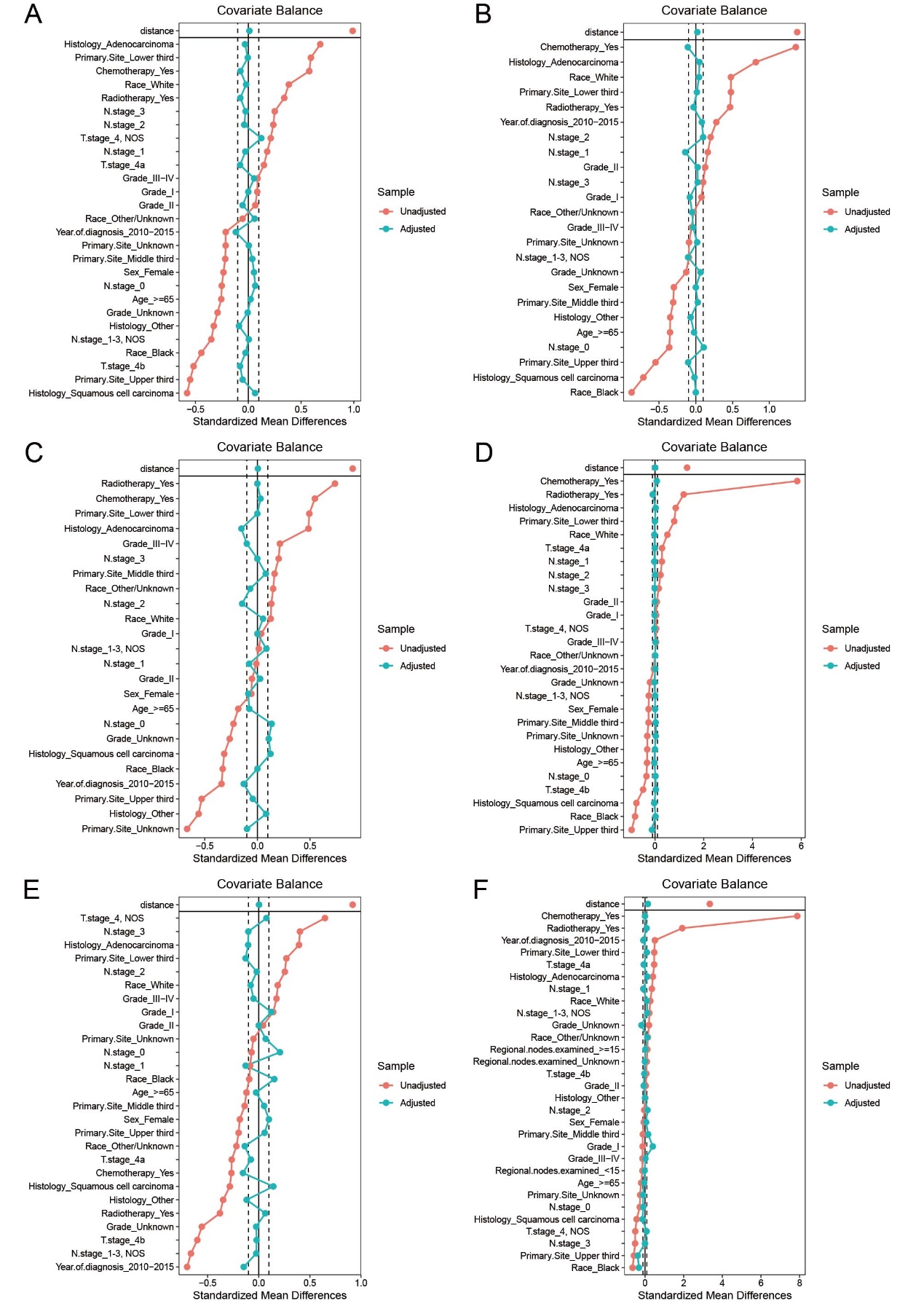


**Supplementary Figure 1** Baseline standardized mean difference before and after PSM for surgery vs. no surgery in T4N0-3M0 EC (A), surgery vs. no surgery in T4aN0-3M0 EC (B), surgery vs. no surgery in T4bN0-3M0 EC (C), Neo + surgery vs. no surgery in T4N0-3M0 EC (D), surgery without Neo vs. no surgery in T4N0-3M0 EC (E), and Neo + surgery vs. surgery without Neo in T4N0-3M0 EC (F). PSM, propensity score matching; EC, esophageal cancer; Neo, neoadjuvant therapy.


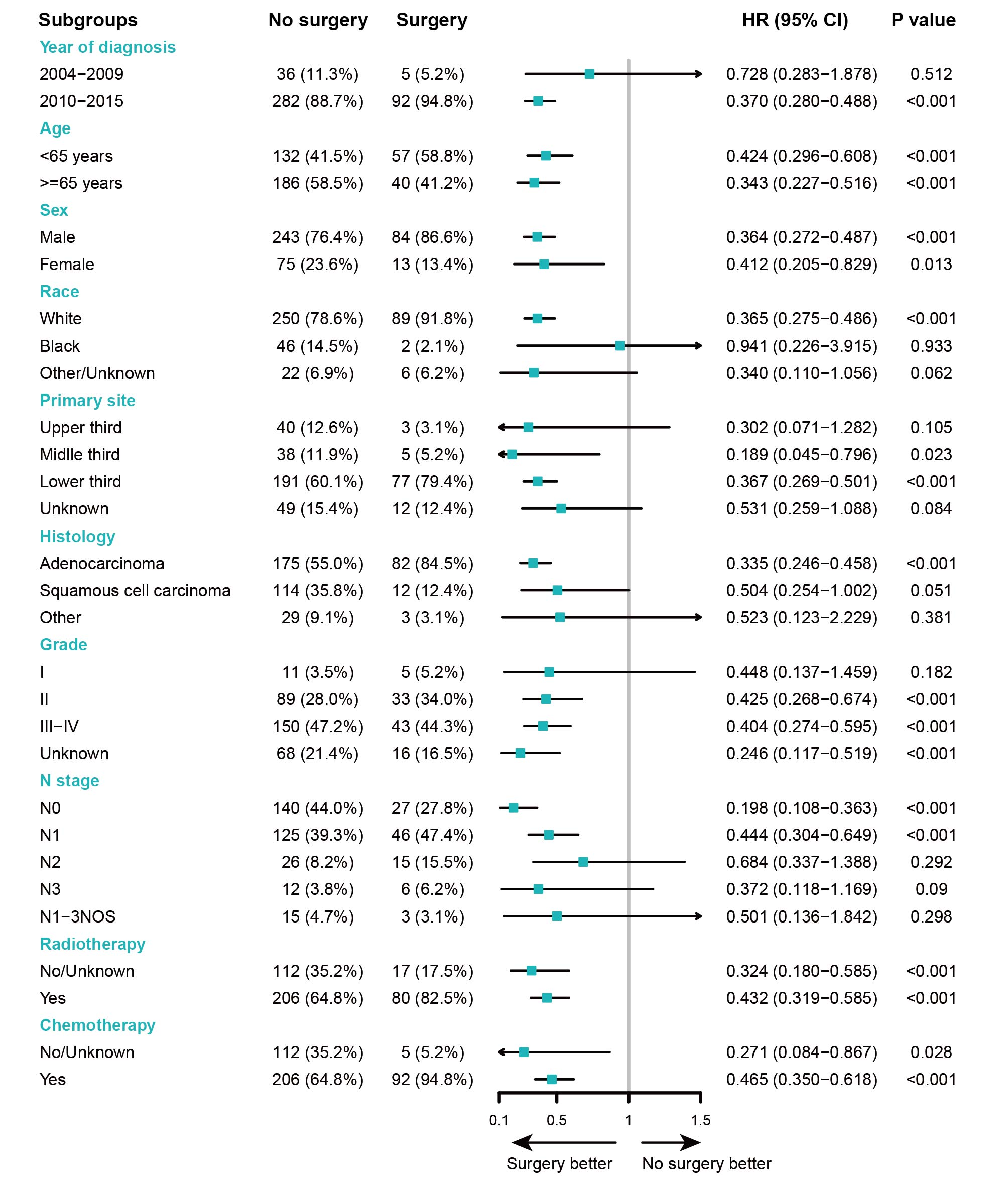


**Supplementary Figure 2** Subgroup analysis of HR for surgery vs. no surgery in OS of stage T4aN0-3M0 EC. HR, hazard ratio; OS, overall survival; EC, esophageal cancer; CI, confidential interval.


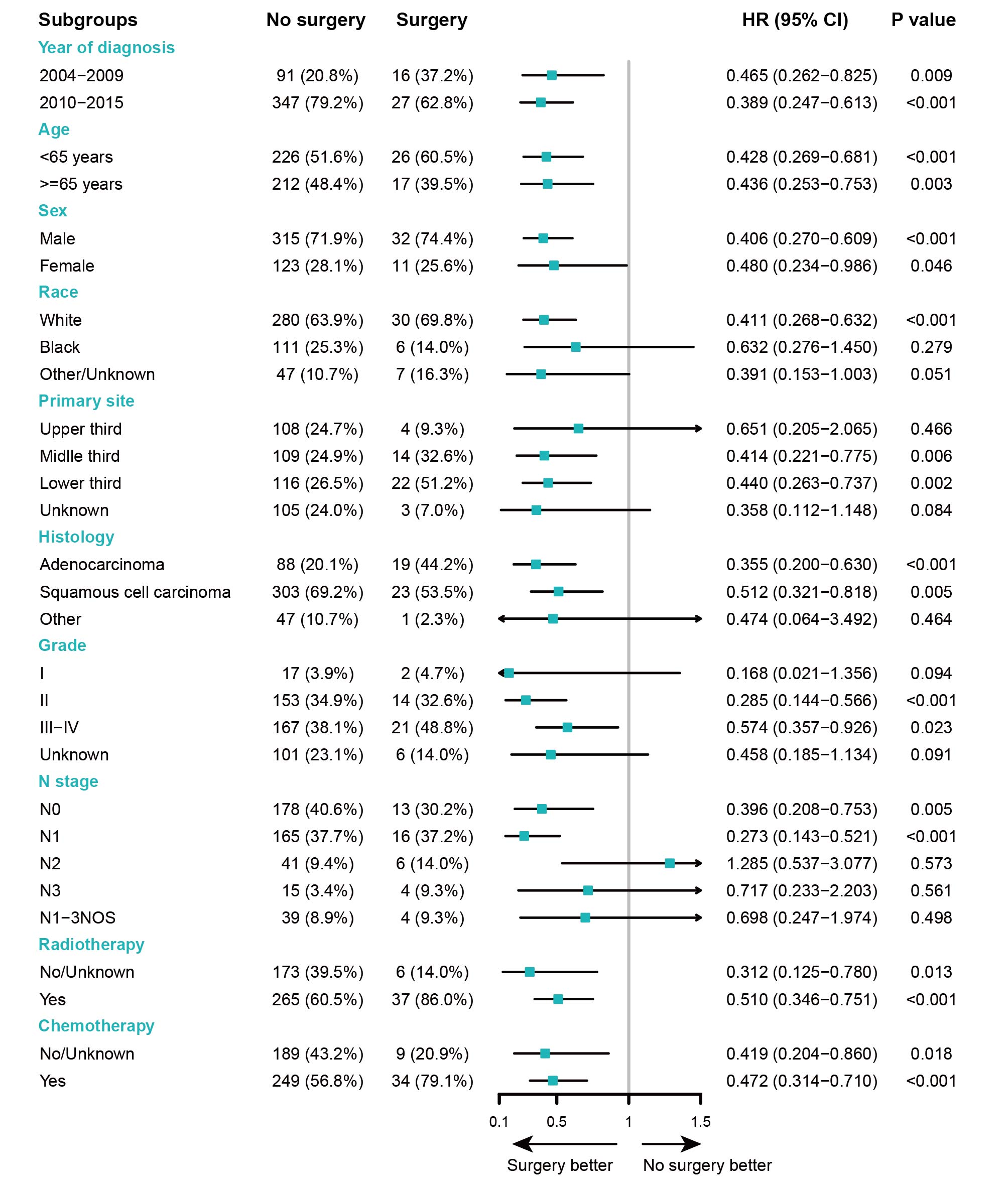


**Supplementary Figure 3** Subgroup analysis of HR for surgery vs. no surgery in OS of stage T4bN0-3M0 EC. HR, hazard ratio; OS, overall survival; EC, esophageal cancer; CI, confidential interval.


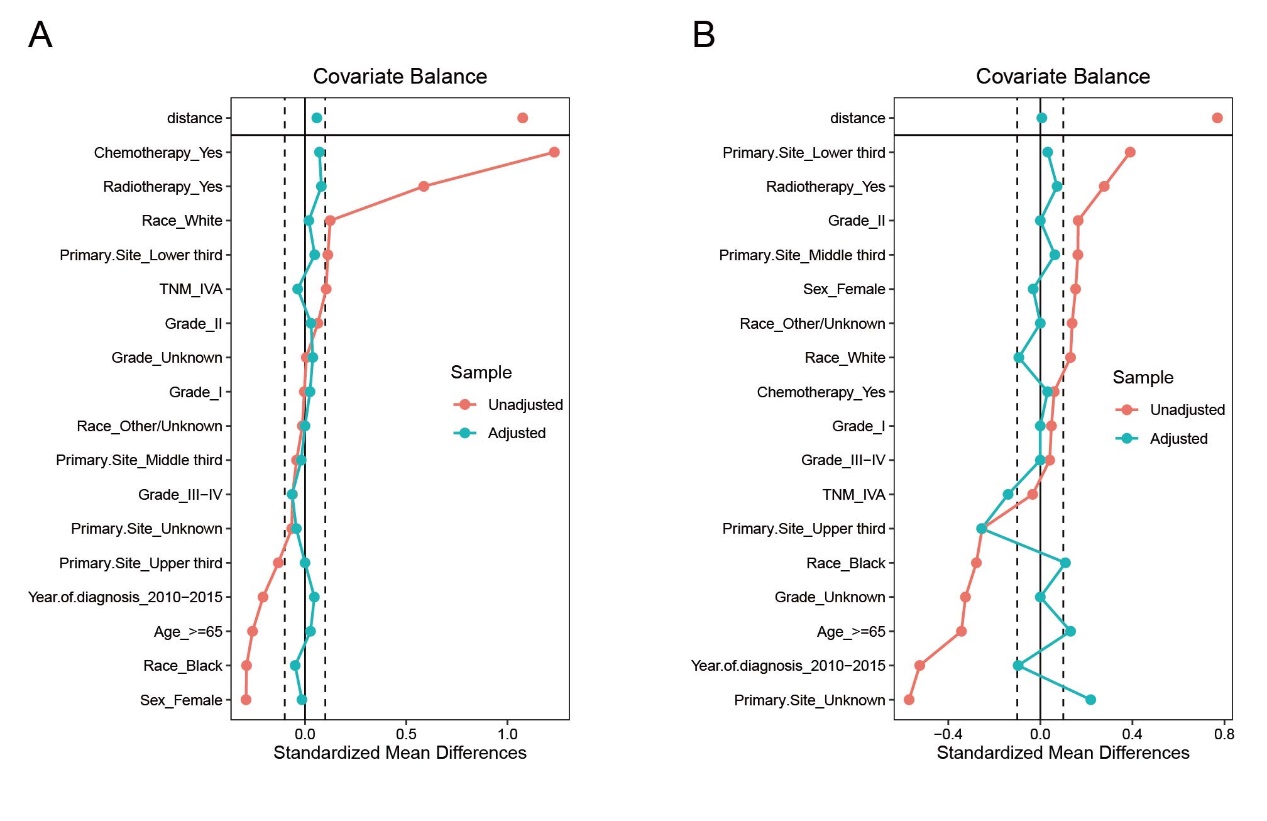


**Supplementary Figure 4** Baseline standardized mean difference before and after PSM for surgery vs. no surgery in IIIB-IVA esophageal adenocarcinoma patients (A), and IIIB-IVA esophageal squamous cell carcinoma patients (B). PSM, propensity score matching.


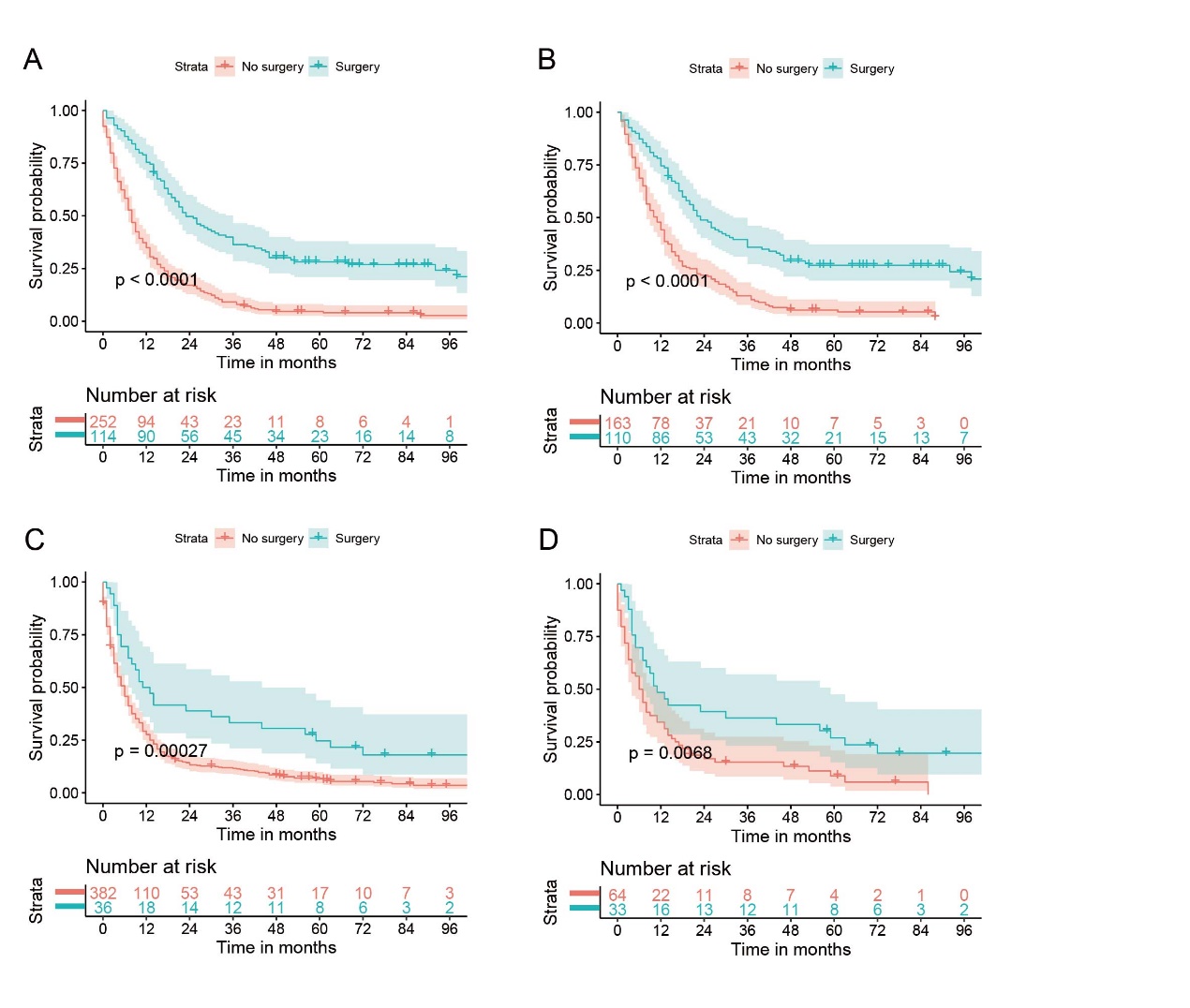


**Supplementary Figure 5** Survival curves of OS for stage IIIB-IVA esophageal adenocarcinoma comparing surgery with no surgery before PSM (A) and after PSM (B), IIIB-IVA esophageal squamous cell carcinoma comparing surgery with no surgery before PSM (C) and after PSM (D). OS, overall survival; PSM, propensity score matching.
